# *HSD3B1* upregulation *via* LRH1 sustains estrogen receptor signaling and promotes endocrine resistance in breast cancer

**DOI:** 10.1016/j.jbc.2025.110405

**Published:** 2025-06-20

**Authors:** Xiuxiu Li, Yoon-Mi Chung, Monaben Patel, Nima Sharifi

**Affiliations:** 1 Desai Sethi Urology Institute, Sylvester Comprehensive Cancer Center, University of Miami Miller School of Medicine, Miami, Florida, USA; 2 Lerner Research Institute, Cleveland Clinic, Cleveland, Ohio, USA

**Keywords:** *HSD3B1*, estrogen synthesis, endocrine resistance, breast cancer

## Abstract

Endocrine resistance is a major challenge in the treatment of estrogen receptor-positive (ER^+^) breast cancer, often leading to disease recurrence and metastasis. 3β-Hydroxysteroid dehydrogenase 1 (3βHSD1, encoded by *HSD3B1*) catalyzes the rate-limiting conversion of dehydroepiandrosterone (DHEA) to androstenedione (AD), the major substrate for aromatase and a key precursor for estrogen biosynthesis. However, the regulation of *HSD3B1* in endocrine-resistant breast cancer remains unclear. We show that long-term estrogen deprivation (LTED) or tamoxifen treatment induces *HSD3B1* expression and enzymatic activity, sustaining DHEA metabolism and ER signaling in resistant ER^+^ breast cancer cells. T47D-LTED and T47D-4OHT cells exhibited increased *HSD3B1* expression and enhanced DHEA metabolism. *HSD3B1* deficiency impaired DHEA-driven survival, confirming its role in endocrine resistance. Mechanistically, we identify liver receptor homolog-1 (LRH1/NR5A2) as a key transcriptional regulator of *HSD3B1*. LRH1 inhibition suppressed *HSD3B1* expression, DHEA metabolism, and ER target gene activation, demonstrating its role in sustaining estrogen synthesis and tumor adaptation. Our findings establish *HSD3B1* as a critical mediator of endocrine resistance and identify LRH1 as an upstream regulator. Targeting *HSD3B1* or LRH1 may offer a new therapeutic strategy to restore endocrine sensitivity in ER^+^ breast cancer.

Estrogen receptor-positive (ER+) breast cancer, which accounts for approximately 70% of breast malignancies, relies on estrogen signaling for growth and survival. Endocrine therapies, including aromatase inhibitors (AIs) and selective estrogen receptor modulators (*e.g.*, tamoxifen) (1), are primarily treatments for ER+ breast cancer. However, intrinsic and acquired resistance to these therapies poses a significant clinical challenge (2, 3), with many patients experiencing disease recurrence and metastasis despite initial responses (4, 5). While mechanisms such as ER mutations and alternative signaling pathways (*e.g.*, HER2, mTOR) are well-characterized drivers of resistance (6, 7, 8, 9), emerging evidence underscores the potential role of adrenal-derived steroid precursors in sustaining intratumoral estrogen synthesis under therapeutic pressure (10, 11, 12).

The enzyme 3β-hydroxysteroid dehydrogenase 1 (3βHSD1, encoded by *HSD3B1*), which catalyzes the initial, irreversible, and rate-limiting step in the conversion of the adrenal-derived steroid dehydroepiandrosterone (DHEA) to androstenedione (AD), the immediate precursor for both androgen and estrogen synthesis (13, 14, 15),is a critical enzymatic gatekeeper that confers tumors with the ability to harness adrenal androgens (16, 17).

A germline polymorphism in *HSD3B1* (1245A > C) distinguishes two functional phenotypes: the adrenal-restrictive genotype (1245A), associated with rapid enzyme degradation, and the adrenal-permissive genotype (1245C), which stabilizes 3βHSD1, enabling enhanced androgen and estrogen precursor synthesis (18). In men with prostate cancer, the adrenal-restrictive *HSD3B1*(1245A) allele limits conversion from DHEA to potent androgens, whereas the adrenal-permissive *HSD3B1*(1245C) allele harbors a 367T missense that stabilizes the enzyme, enables potent androgen synthesis, and leads to more rapid development of resistance to androgen deprivation therapy and next-generation hormonal therapies, thus shortening overall survival (19, 20, 21, 22, 23, 24, 25).

Emerging evidence suggests a similar role in breast cancer. Notably, recent clinical studies suggest that the adrenal-permissive genotype might be contributory to postmenopausal ER+ breast cancer (12). Additionally, a recent study reported that breast cancer patients carrying the homozygous adrenal-permissive genotype had a higher risk of distant metastases and mortality, reinforcing the role of intratumoral steroid metabolism in disease progression (26). Other studies point toward a broader role for *HSD3B1* inheritance in determining hormone-dependent vs. hormone-independent subtypes of breast and endometrial cancer (27). Despite these advances, the role of *HSD3B1* under endocrine therapy pressure and its mechanistic link to therapy resistance remain poorly understood.

Liver receptor homolog-1 (LRH1/NR5A2), a nuclear receptor critical for steroidogenic gene expression, is implicated in prostate cancer progression through driving steroidogenic genes (28, 29, 30), but is poorly studied in breast cancer. Here, we investigate whether long-term estrogen deprivation (LTED) or tamoxifen exposure induces *HSD3B1* expression *via* LRH1, enabling DHEA-driven ER reactivation. Using ER+ breast cancer models with distinct *HSD3B1* genotypes, we demonstrate that therapy-resistant cells upregulate 3βHSD1 activity to sustain survival and ER signaling. These findings identify 3βHSD1 and LRH1 as actionable targets to restore therapeutic sensitivity in endocrine-resistant breast cancer.

## Results

### Enhanced 3βHSD1 expression and activity following long-term estrogen deprivation (LTED) or 4-hydroxy-Tamoxifen (4OHT) treatment

**Figure 1:**
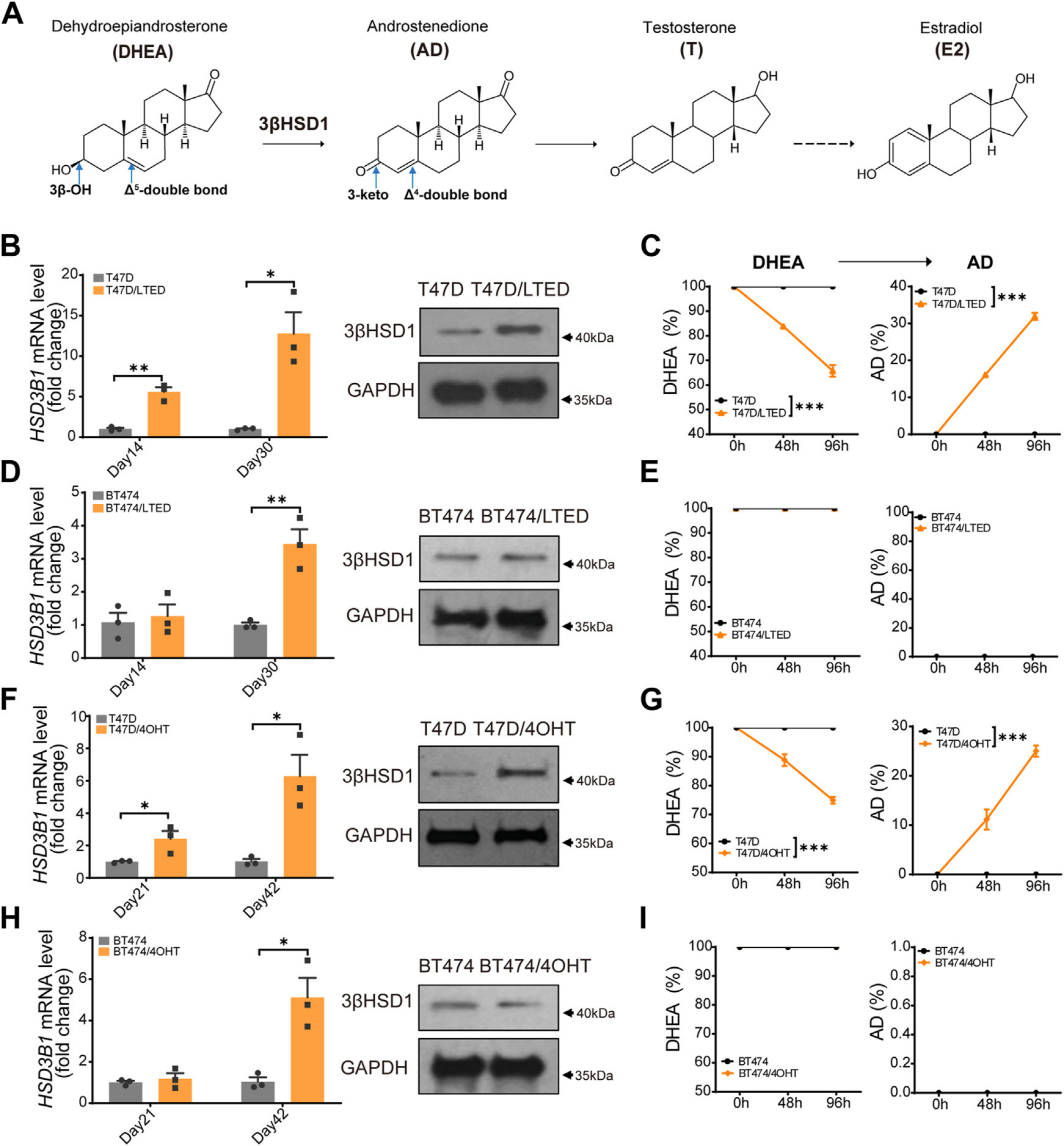
**Enhanced 3βHSD1 expression and activity in long-term estrogen deprivation (LTED) or 4OHT-treated T47D cells**. *A*, 3β-hydroxysteroid dehydrogenase-1 (3βHSD1) converts dehydroepiandrosterone (DHEA) to androstenedione, which is subsequently converted to estrogens. *B and D*, T47D or BT474 cells were cultured in 10% charcoal-stripped serum (LTED) RPMI-1640 medium for the indicated duration, and *HSD3B1* mRNA levels were assessed by qPCR and protein levels were assessed by Western blot. *C and E*, to assess 3βHSD1 activity, T47D, T47D-LTED or BT474, BT474-LTED cells were treated with [^3^H]-DHEA, and metabolites were analyzed by high-performance liquid chromatography (HPLC). *F and H*, T47D or BT474 cells were treated with 2 μM 4-hydroxy tamoxifen (4OHT) for indicated duration, followed by *HSD3B1* expression analysis by qPCR and Western blot. *G and I*, to assess 3βHSD1 activity, T47D, T47D-4OHT or BT474, BT474-4OHT cells were treated with [^3^H]-DHEA, and metabolites were analyzed by high-performance liquid chromatography (HPLC). For all *panels*, unless otherwise noted, error bars represent the SEM; *p* values were calculated using un-paired two-tailed t tests. ∗*p* < 0.05. ∗∗*p* < 0.01. ∗∗∗*p* < 0.001.

To investigate the role of *HSD3B1* in hormone therapy resistance, we analyzed the *HSD3B1* genotype across multiple ER-positive breast cancer cell lines to determine whether they carried the adrenal-permissive or adrenal-restrictive allele, following a method as reported previously (31). Our results showed that T47D cells are homozygous for the adrenal-permissive allele, while BT474 cells are homozygous for the adrenal-restrictive allele. Additionally, MCF7 and ZR-75-1 cells were found to be heterozygous. Based on these findings, we selected T47D (permissive) and BT474 (restrictive) cells for further investigation. T47D (adrenal-permissive *HSD3B1* genotype) and BT-474 (adrenal-restrictive *HSD3B1* genotype) were subjected to long-term estrogen deprivation (LTED) or 4OHT treatment; quantitative PCR (qPCR) and Western blot analysis revealed a significant increase in *HSD3B1* mRNA and protein expression in T47D-LTED and T47D-4OHT cells compared to parental controls (Fig. 1, A, B and F). In BT474 cells, LTED or 4OHT treatment induced an increase in *HSD3B1* mRNA levels but not protein levels, consistent with its adrenal-restrictive genotype, which leads to lower protein stability (Fig. 1, *D* and *H*).

To determine whether the increase in *HSD3B1* expression correlates with enhanced enzymatic activity, we performed [^3^H]-DHEA metabolism assays using high-performance liquid chromatography (HPLC). The conversion of DHEA to androstenedione (AD) was significantly increased in T47D-LTED and T47D-4OHT cells, indicating enhanced 3βHSD1 metabolic activity (Fig. 1, *C* and *G*), In contrast, BT474-LTED and BT474-4OHT cells did not show increased DHEA metabolism (Fig. 1, *E* and *I*), consistent with their lower 3βHSD1 protein expression. Furthermore, both mRNA and protein levels of *HSD3B1* are increased following long-term estrogen deprivation or 4OHT treatment in MCF7 cells, suggesting that upregulation of *HSD3B1* in response to endocrine therapy is not limited to homozygous contexts (Fig. S1*A*). Moreover, we observed that long-term treatment with an aromatase inhibitor (letrozole) also led to increased *HSD3B1* mRNA expression, suggesting that *HSD3B1* upregulation is a general feature of endocrine therapy resistance (Fig. S1*B*). While we examined the expression levels of ERα, ERβ, and aromatase in T47D, T47D-LTED, and T47D-4OHT cells, our results showed no significant differences in either mRNA or protein levels after LTED or 4OHT treatment (Fig. S1, *C* and *D*).

### Enhanced 3βHSD1 activity promotes cell survival under DHEA treatment

**Figure 2:**
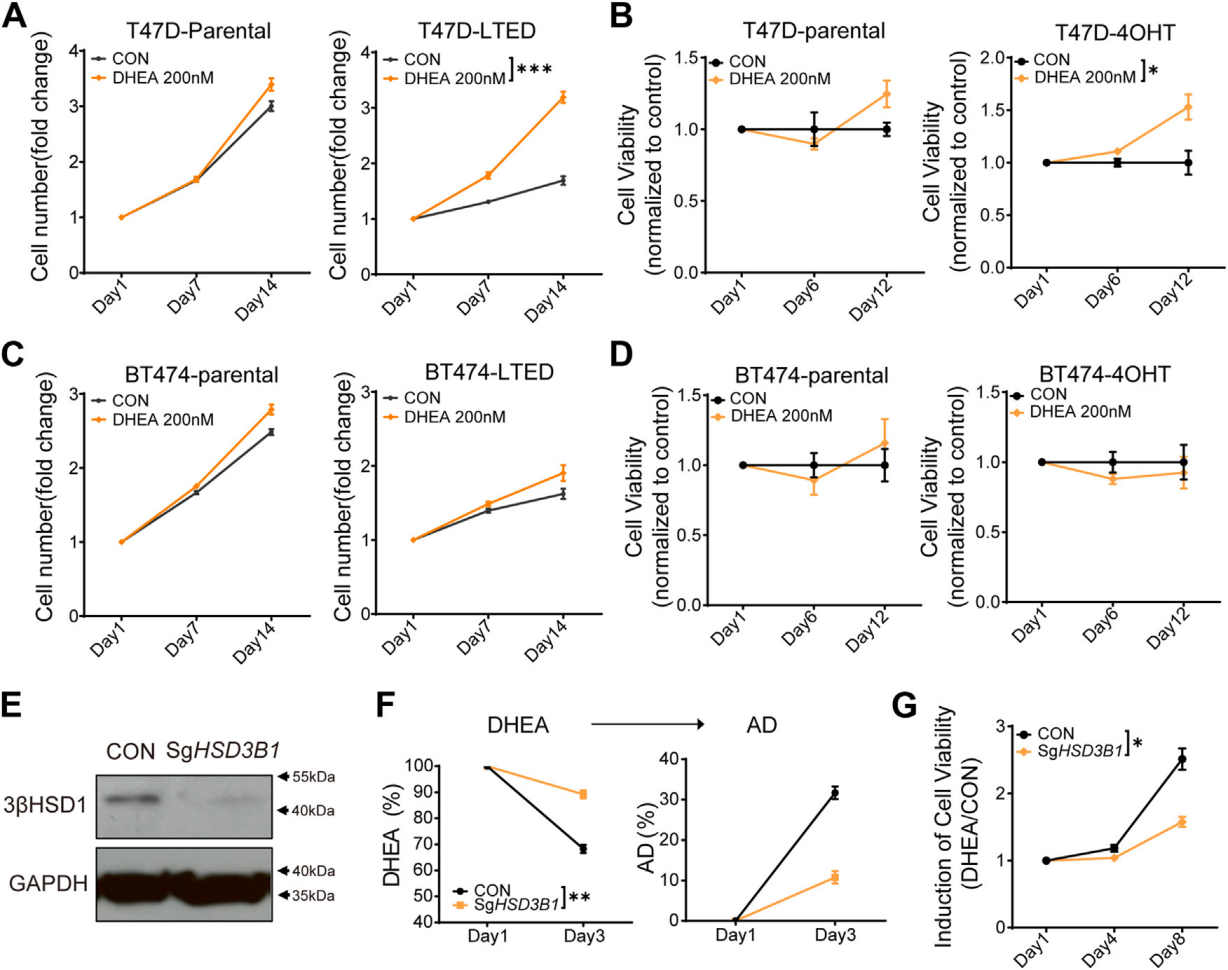
**Enhanced 3βHSD1 activity promotes cell survival in long-term estrogen deprivation (LTED) or 4OHT-treated T47D cells**. *A*, T47D cells were cultured in 10% CSS medium for more than 14 days, treated with 100 nM DHEA, and cell surival was assessed using trypan *blue* staining. *B*, T47D cells were treated with 2 μM tamoxifen for over 30 days, followed by 100 nM DHEA treatment, and cell viability was assessed. *C and D*, BT474 parental and LTED/TAM cells were cultured and treated as (*A*) and (*B*), followed by cell survival/cell viability assessment. *E*, Stable T47D cell lines with *HSD3B1* gRNA or control gRNA were generated, and 3βHSD1 protein expression was analyzed by Western blot. *F,* stable T47D cell *lines* with *HSD3B1* gRNA or control gRNA were cultured in 10% CSS for 21 days, treated with [^3^H]-DHEA, and steroid metabolism was analyzed by HPLC. *G*, stable T47D cell *lines* with *HSD3B1* gRNA or control gRNA were cultured in 10% CSS for 21 days, followed by 100 nM DHEA treatment, and cell viability was assessed. Data represent mean ± SEM of three independent experiments. *p* values were calculated using un-paired two-tailed t tests. ∗*p* < 0.05. ∗∗*p* < 0.01. ∗∗∗*p* < 0.001.

To evaluate the functional significance of increased 3βHSD1 expression and activity, we investigated whether DHEA treatment supports cell survival. Parental T47D, T47D-LTED, and T47D-4OHT cells were treated with DHEA, and measured cell growth and cell viability were measured using trypan blue staining and cell viability assays. The results showed that DHEA significantly promoted the cell growth and viability of T47D-LTED and T47D-4OHT cells, whereas parental T47D cells did not exhibit a significant response to DHEA (Fig. 2, *A* and *B*). In contrast, BT474-LTED and BT474-4OHT cells did not show increased cell growth and viability in response to DHEA, consistent with their lack of 3βHSD1 metabolic activity (Fig. 2, *C* and *D*).

To confirm that 3βHSD1 enzymatic activity was responsible for the observed cell survival advantage, we generated stable T47D cell lines with CRISPR-mediated knockout of *HSD3B1* and validated the knockout by Western blot analysis (Fig. 2*E*). Notably, we determined that there is incomplete loss of 3βHSD1 expression, which is probably attributable to this being a pool of cells subjected to CRISPR. Nevertheless, these *HSD3B1*-deficient cells were then cultured in LTED medium for 21 days, treated with [^3^H]-DHEA, and analyzed by HPLC to confirm the loss of 3βHSD1 metabolic activity (Fig. 2*F*). Importantly, genetic deletion of *HSD3B1* significantly attenuated DHEA-induced cell viability, demonstrating that *HSD3B1*-mediated DHEA metabolism supports cell survival under estrogen-deprived conditions (Fig. 2*G*).

### Enhanced 3βHSD1 activity promotes estrogen receptor (ER) signaling

**Figure 3:**
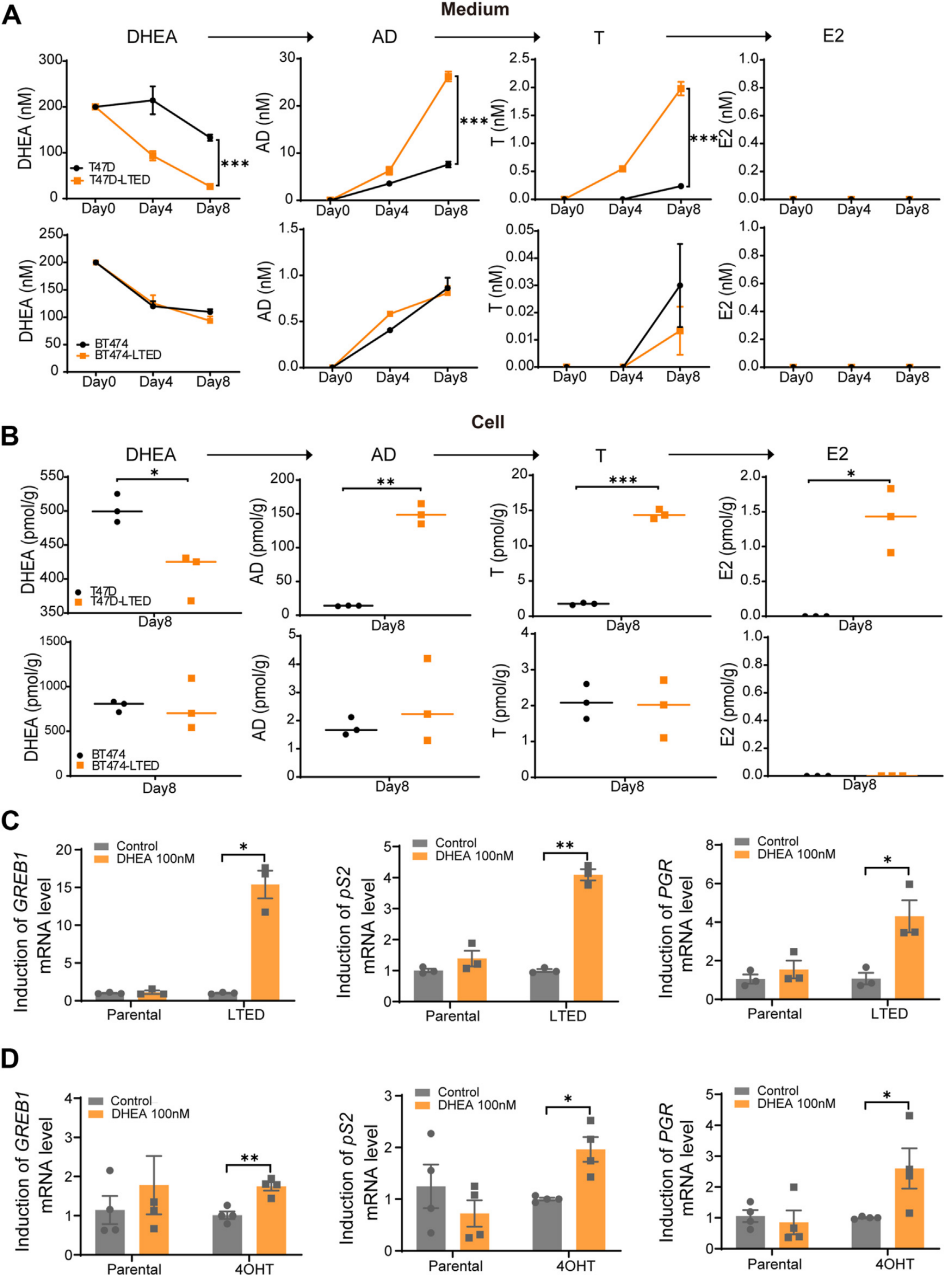
**Enhanced 3βHSD1 activity promotes ER signaling in long-term estrogen deprivation (LTED) or 4OHT-treated T47D cells**. *A and B*, T47D, T47D-LTED, BT474, BT474- LTED cells were starved with RPMI-1640 medium containing 10% charcoal-stripped fetal bovine serum; 48 h later, 200 nM DHEA was added, cells were cultured for the indicated times, medium and cells were collected, and mass spectrometry was performed to detect the steroid level. *C*, T47D parental and LTED cells were cultured in 10% CSS for 21 days, treated with 100 nM DHEA for 7 days, followed by qPCR analysis of ER target genes (*GREB1*, *pS2*, and *PGR*). *D*, T47D parental and long-term tamoxifen-treated (T47D-4OHT) cells were treated with 100 nM DHEA under 1 μM 4-hydroxy tamoxifen (4OHT) for 7 days, followed by qPCR analysis of ER target gene expression. Expression was normalized to RPLP0. Data is represented as mean ± SEM from three independent biological replicates (unpaired two-tailed *t* test). ∗*p* < 0.05. ∗∗*p* < 0.01. ∗∗∗*p* < 0.001.

Since 3βHSD1 catalyzes DHEA conversion into estrogen precursors, we next evaluated whether its increased activity leads to enhanced estrogen production and ER signaling activation. DHEA treatment in T47D-LTED cells led to a significant increase in androstenedione (AD), testosterone (T), and estradiol (E2) levels from DHEA, as detected by mass spectrometry. In contrast, BT474-LTED cells showed no significant difference in androstenedione (AD), testosterone (T), and estradiol (E2) production. (Fig. 3, *A* and *B*).

T47D parental and LTED cells were treated with 100 nM DHEA for 7 days. We assessed the expression of ER target genes (*GREB1*, *pS2*, and *PGR*) by qPCR. The results showed that DHEA treatment significantly increased ER target gene expression in T47D-LTED cells compared to parental T47D cells, indicating enhanced ER transcriptional activity (Fig. 3*C*). Similarly, DHEA treatment also upregulated ER target gene expression in long-term tamoxifen-treated T47D-4OHT cells (Fig. 3*D*). These findings suggest that increased 3βHSD1 activity enhances estrogen biosynthesis, sustaining ER signaling in therapy-resistant cells.

### LRH1 drives *HSD3B1* induction in therapy-resistant cells

**Figure 4:**
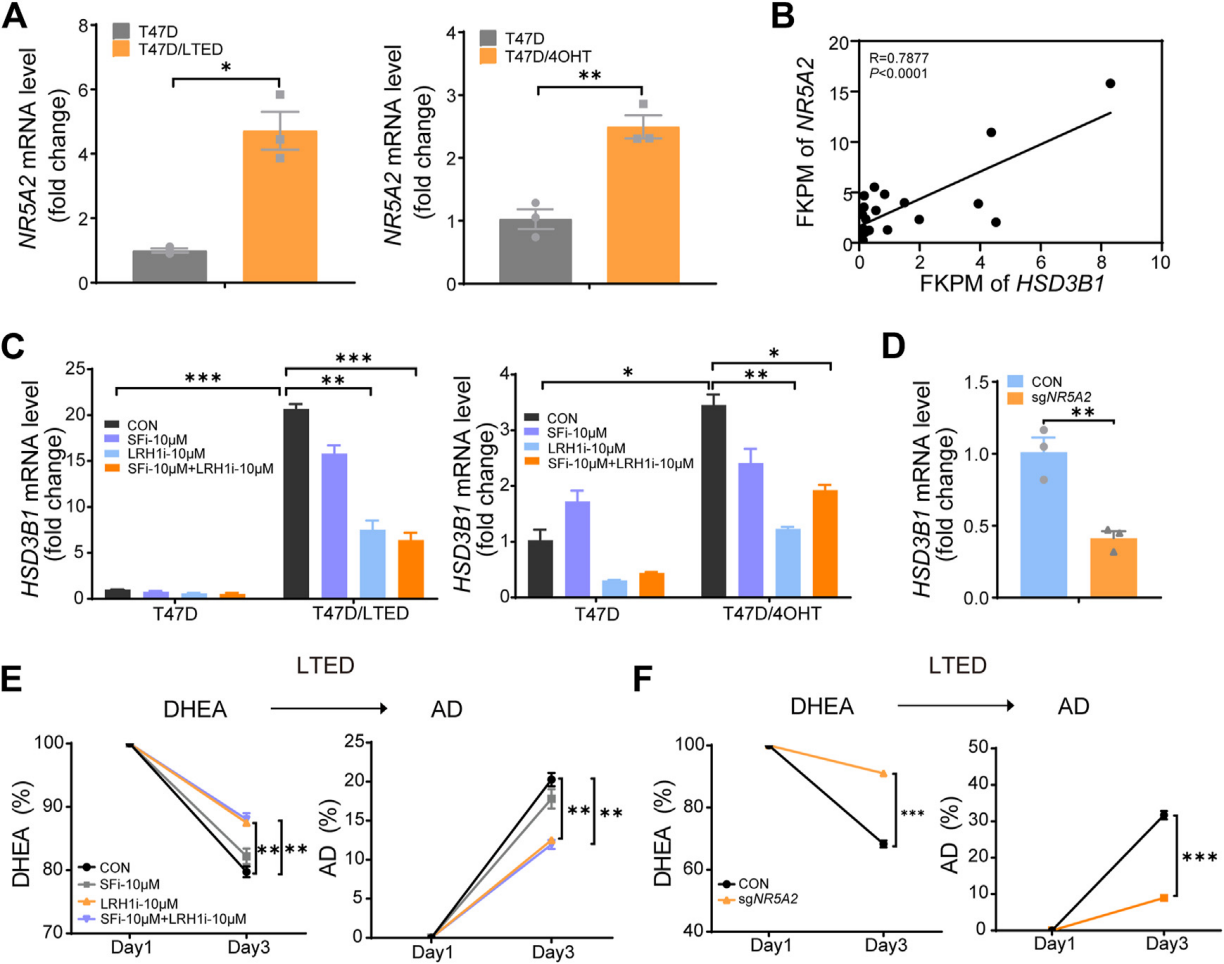
**LRH1, but not SF1, regulates *HSD3B1* expression and steroid metabolism in endocrine-resistant T47D cells**. *A*, NR5A2 (LRH1) expression was analyzed in T47D and BT474 cells following LTED or long-term tamoxifen treatment by qPCR. *B*, RNA-seq data from the POP trial (NCT02008734) was analyzed for correlation between *HSD3B1* and LRH1 expression. *C*, T47D, T47D-LTED, and T47D-TAM cells were treated with SF1 or LRH1 inhibitors for 48 h, followed by qPCR analysis of *HSD3B1* expression. *D*, stable T47D cell lines expressing NR5A2 gRNA or control gRNA were generated, and *HSD3B1* expression was analyzed by qPCR. *E*, T47D-LTED cells were treated with SF1 or LRH1 inhibitors for 12 h, followed by [^3^H]-DHEA metabolism analysis by HPLC. *F*, NR5A2 gRNA or control T47D cell lines were cultured in RPMI-1640 medium containing 10% charcoal-stripped fetal bovine serum for 21 days, treated with [^3^H]-DHEA, and steroid metabolism was assessed by HPLC. Data is represented as mean ± SEM from three independent biological replicates (unpaired two-tailed *t* test). ∗*p* < 0.05. ∗∗*p* < 0.01. ∗∗∗*p* < 0.001.

Mechanistically, we explored transcriptional regulation of *HSD3B1* in LTED and 4OHT-resistant cells. We examined the involvement of liver receptor homolog-1 (LRH1/NR5A2) and steroidogenic factor-1 (SF1/NR5A1), two known transcriptional regulators of steroidogenic enzymes. qPCR and Western Blot analysis of T47D cells following LTED or long-term 4OHT treatment revealed that LRH1 mRNA and protein expression was significantly upregulated in T47D-LTED and T47D-4OHT cells, while SF1 expression remained at a very low level (Fig. 4*A* and Fig. S2*A*). To determine the clinical relevance of these findings, we reanalyzed processed RNA-seq data from hormone therapy–treated breast cancer patients enrolled in the POP trial (NCT02008734), as published in the supplementary materials of Guerrero-Zotano et al. (32). We found a positive correlation between *HSD3B1* and *NR5A2* expression in hormone therapy-treated breast cancer patients (Fig. 4*B*).

To directly test whether LRH1 regulates *HSD3B1* expression, T47D, T47D-LTED, and T47D-4OHT cells were treated with SF1 or LRH1 inhibitors for 48 h, followed by qPCR analysis of *HSD3B1* expression. The results showed that LRH1 inhibition significantly reduced *HSD3B1* expression, whereas SF1 inhibition had no effect (Fig. 4*C*). Furthermore, CRISPR-mediated knockout of *NR5A2* (LRH1) in T47D cells (Fig. S3*B*) also led to a marked reduction in *HSD3B1* expression, and LRH1 overexpression significantly increased *HSD3B1* levels (Fig. S2*D*), further confirming LRH1's role in *HSD3B1* regulation (Fig. 4*D*). To assess the broader applicability of LRH1-mediated regulation of *HSD3B1*, we tested whether LRH1 alters *HSD3B1* levels in MCF7 cells. Our findings showed that LRH1 inhibition significantly decreases *HSD3B1* expression, while LRH1 overexpression increases it, supporting a conserved regulatory mechanism across multiple breast cancer models (Fig. S2, *B* and *C*). To determine whether LRH1 directly binds to the *HSD3B1* promoter, we performed chromatin immunoprecipitation (ChIP) assays. Two high-scoring LRH1 binding motifs (AACCCAAAGGTCACT and GAGTACATGGCCAGA) were identified within the proximal promoter region (−130 to −60 bp upstream of the transcription start site) using the JASPAR database. Three primer sets spanning this region were designed, and ChIP-qPCR was conducted using the SimpleChIP Sonication Kit. These analyses demonstrated direct LRH1 occupancy at the *HSD3B1* promoter, supporting transcriptional regulation by LRH1(Fig. S2*E*).

To assess whether LRH1 influences DHEA metabolism, T47D-LTED, T47D-4OHT cells were treated with SF1 or LRH1 inhibitors for 12 h, followed by [^3^H]-DHEA metabolism analysis using HPLC. The results showed that LRH1 inhibition significantly suppressed DHEA metabolism, whereas SF1 inhibition had no effect (Figs. 4*E*, S3*A*). Similarly, T47D-LTED, T47D-4OHT cells with LRH1 knockout showed a reduction in DHEA metabolism compared to control cells, further supporting the role of LRH1 in *HSD3B1*-mediated steroidogenesis (Figs. 4*F*, S3*C*).

### LRH1 blockade impedes the expression of estrogen-regulated genes and breast cancer cell viability

**Figure 5:**
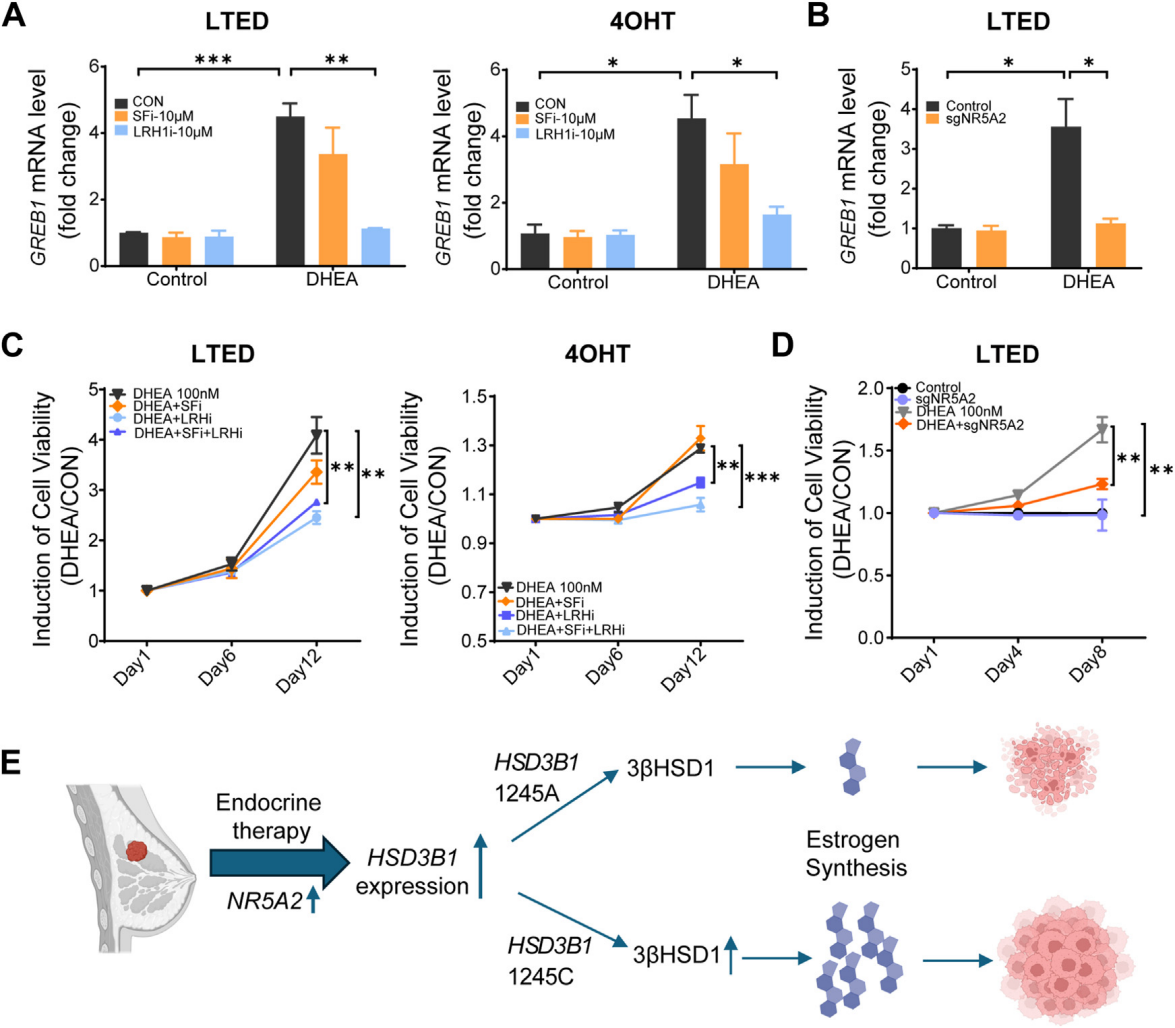
**NR5A2 blockade impedes expression of estrogen-regulated genes and breast cancer cell viability**. *A*, T47D-LTED and T47D-4OHT cells were treated with SF1 or LRH1 inhibitors (10 μM) for 12 h, followed by DHEA treatment (100 nM) for 7 days, and qPCR analysis of ER target gene expression. *B*, NR5A2 gRNA or control T47D cell lines were cultured in RPMI-1640 medium containing 10% charcoal-stripped fetal bovine serum for 21 days, followed by DHEA treatment for 7 days, and qPCR analysis of ER target gene expression. *C*, T47D-LTED, and T47D-4OHT cells were pre-treated with SF1 or LRH1 inhibitors (10 μM) for 12 h, followed by DHEA treatment for the indicated days, and cell viability was assessed. *D*, NR5A2 gRNA or control T47D cell lines were cultured in RPMI-1640 medium containing 10% charcoal-stripped fetal bovine serum for 21 days, followed by DHEA treatment (100 nM) for the indicated days, and cell viability was assessed. Data represent mean ± SEM from three independent biological replicates (unpaired two-tailed *t* test). *E*, model. Our findings indicate that *HSD3B1* upregulation in response to LTED and tamoxifen treatment enhances DHEA metabolism, sustaining ER signaling and promoting cell survival, enabling tumors to exploit adrenal androgens for survival under therapy pressure. This upregulation is partially regulated by LRH1. Data are represented as mean ± SEM from three independent biological replicates (unpaired two-tailed *t* test). ∗*p* < 0.05. ∗∗*p* < 0.01. ∗∗∗*p* < 0.001.

To further investigate the functional role of LRH1 in endocrine-resistant breast cancer, we treated T47D-LTED and T47D-4OHT cells with a specific LRH1 inhibitor and evaluated its effects on ER-dependent gene expression and cell viability. T47D-LTED and T47D-4OHT cells were pre-treated with either an SF1 or LRH1 inhibitor for 12 h, followed by 100 nM DHEA treatment for 7 days qPCR analysis revealed that LRH1 inhibition, but not SF1 inhibition, significantly reduced DHEA-induced ER target gene expression, while not affect control or estradiol-induced expression (Fig. 5*A*, Fig. S4*A*). Similarly, CRISPR-mediated LRH1 knockout produced comparable results (Figs. 5*B*, S4*B*), indicating that LRH1 plays a key role in maintaining ER signaling by regulating 3βHSD1-mediated estrogen synthesis.

Next, we examined the impact of LRH1 inhibition on cell viability. T47D-LTED and T47D-4OHT cells were pre-treated with SF1 or LRH1 inhibitors for 12 h, followed by 100 nM DHEA treatment for the indicated days. Cell viability was assessed using the CellTiter-Glo Luminescent Cell Viability Assay. The results demonstrated that LRH1 inhibition significantly reduced DHEA-induced cell viability (Fig. 5*C*), whereas SF1 inhibition had no significant effect. By contrast, LRH1 inhibition by ML180 (IC50 = 3.7 μM) (33) had no significant effect on control or estradiol-induced cell viability (Fig. S4, *C* and *D*). Similarly, CRISPR-mediated LRH1 knockout also impaired DHEA-driven viability (Figs. 5*D*, S4*E*). To test whether LRH1 upregulation is sufficient to drive resistance, we overexpressed LRH1 in T47D cells and assessed downstream effects. LRH1 overexpression significantly enhanced DHEA-induced ER target gene expression and cell viability (Fig. S4*F*). This further confirms that LRH1 facilitates DHEA metabolism to estrogens and supports breast cancer cell survival under endocrine therapy pressure by upregulating *HSD3B1* expression.

Collectively, our findings suggest that *HSD3B1* is upregulated in LTED and tamoxifen-treated breast cancer cells, enhancing DHEA metabolism, sustaining ER signaling, and promoting cell survival. This process is at least partially regulated by LRH1 (Fig. 5*E*). Therefore, targeting LRH1-regulated 3βHSD1 may represent a potential therapeutic strategy to overcome endocrine resistance in breast cancer.

## Discussion

Endocrine therapy resistance is a major challenge in the treatment of ER^+^ breast cancer, and our study provides new insights into how *HSD3B1* upregulation following long-term estrogen deprivation (LTED) and tamoxifen treatment supports cell survival and sustains ER signaling, ultimately contributing to therapy resistance.

We establish *HSD3B1* as a critical mediator of endocrine resistance in ER+ breast cancer, with genotype-dependent regulation and LRH1-driven transcriptional control. In adrenal-permissive T47D cells, LTED or tamoxifen treatment robustly increased *HSD3B1* mRNA and protein levels, enhancing DHEA-to-AD conversion and estrogen synthesis. We also determined whether long-term aromatase inhibitor (AI) treatment upregulates *HSD3B1*. As expected, prolonged AI treatment also led to a significant increase in *HSD3B1* expression, further supporting its role in adaptive steroidogenesis under endocrine therapy pressure. These results align with clinical observations where the adrenal-permissive genotype correlates with higher distant metastatic recurrence and breast cancer-specific mortality in postmenopausal ER+ patients (26). Moreover, we demonstrated that both mRNA and protein levels of *HSD3B1* are increased following long-term estrogen deprivation or 4OHT treatment in MCF7 cells, suggesting that upregulation of *HSD3B1* in response to endocrine therapy is not limited to homozygous contexts.

The functional significance of 3βHSD1 was evident in rescue experiments: DHEA restored viability in resistant cells, while genetic inhibition of 3βHSD1 reversed this effect. Notably, DHEA also activated ER target genes, confirming that intracrine steroidogenesis sustains ER signaling. Our work mechanistically links *HSD3B1* induction to LRH1 in breast cancer steroidogenesis. LRH1 upregulation in resistant cells, coupled with its correlation with *HSD3B1* in clinical datasets, underscores its role in adaptive steroidogenesis. The absence of SF1 involvement contrasts with its established role in prostate cancer, highlighting tissue-specific regulatory networks (28, 34).

Our findings propose two strategies to counteract resistance (1): direct inhibition of 3βHSD1 enzymatic activity and (2) blockade of LRH1 to prevent 3βHSD1 induction. Small-molecule inhibitors targeting 3βHSD1, which abrogate DHEA-driven survival, warrant further discovery. Similarly, LRH1 antagonists could preempt adaptive steroidogenesis, particularly in tumors with adrenal-permissive genotypes. Combining these agents with endocrine therapies may delay or reverse resistance, though genotype-stratified trials will be essential.

However, our study lacks *in vivo* animal experiments and more comprehensive analyses in patients undergoing endocrine therapy. Next steps should focus on validating these findings in patients receiving endocrine therapy to determine the clinical relevance and its potential as a biomarker or therapeutic target in endocrine-resistant breast cancer.

## Conclusion

Our findings indicate that 3βHSD1 upregulation in response to LTED and tamoxifen treatment enhances DHEA metabolism, sustaining ER signaling and promoting cell survival, enabling tumors to exploit adrenal androgens for survival under therapy pressure. This upregulation is partially regulated by LRH1, suggesting that targeting 3βHSD1 or LRH1 may provide a novel therapeutic strategy to overcome endocrine therapy resistance in ER-positive breast cancer.

## Experimental procedures

### Cell lines and constructs

All cell lines were authenticated by short tandem repeat (STR) profiling performed by the institutional core facility and tested negative for *mycoplasma* contamination using MycoStrip 100 (rep-mysnc-100, Invivogen). ER-positive breast cancer cell lines T47D and BT-474 were purchased from the American Type Culture Collection (ATCC) and maintained in RPMI-1640 medium supplemented with 10% fetal bovine serum (FBS) and 1% penicillin-streptomycin at 37 °C in a humidified 5% CO_2_ atmosphere. 293T cells, MCF7, and ZR-75-1 cells were purchased from ATCC and cultured in DMEM containing 10% fetal bovine serum. LTED cells were maintained in RPMI-1640 medium with 10% charcoal-stripped serum, while 4OHT cells were cultured in RPMI-1640 medium supplemented with 1 to 2 μM 4-hydroxytamoxifen.

A guide RNA sequence for targeting *HSD3B1* 5′-CGTTTATACTAGCAGAAAGGC-3′ was designed and cloned; constructs of sgRNA targeting NR5A2 (5′-CGTTTATACTAGCAGAAAGGC-3′) were purchased from Applied Biological Materials. Virus was produced using the LentiCRISPRv2 protocol (35). Next, T47D cells were infected with the concentrated virus for 24 h with the addition of polybrene (10 μg/ml), followed by selection with puromycin (1 μg/ml) for ∼2 weeks.

### Antibodies, chemicals, and reagents

#### Antibodies

Mouse monoclonal antibodies against 3βHSD1(1:2000, ab55268) were purchased from Abcam. Rabbit monoclonal antibodies against NR5A2 (1:1000, 22460-1-AP), were ordered from Proteintech. Rabbit polyclonal antibodies against GAPDH (1:5000, 14C10), Rabbit polyclonal antibodies against Estrogen Receptor α (1:1000, #8644) (36, 37), Rabbit polyclonal antibodies against Estrogen Receptor β (1:1000, #89954) (38) were obtained from Cell Signaling Technology.

#### Chemical

NR5A2 inhibitor ML180 (SR1848) was purchased from Selleckchem. NR5A1 inhibitor SID 7969543 (HY-107404) was purchased from MedChemExpress. [^3^H]-labeled DHEA (100 nM, 300,000–600,000 cpm) was purchased from PerkinElmer, and steroids were purchased from Steraloids.

#### Reagents

Puromycin (A1113803) and hygromycin (10,687,010) were bought from ThermoFisher Scientific. DNA transfection reagent FuGENE HD (E2311) was purchased from Promega. GelCode Blue Stain Reagent (24,590) was obtained from Pierce.

### RNA extraction and quantitative PCR (qPCR)

Total RNA was extracted with GenElute Mammalian Total RNA miniprep kit (Sigma-Aldrich), and 1 μg RNA was reverse-transcribed to complementary DNA (cDNA) with the iScript cDNA Synthesis Kit (Bio-Rad). An ABI 7500 Real-Time PCR instrument (Applied Biosystems) was used to perform quantitative PCR (qPCR) analysis, using iTaq Fast SYBR Green Supermix with ROX (Bio-Rad) in 96-well plates at a final reaction volume of 10 μl. The qPCR analysis was carried out in triplicate with the following primer sets:

*HSD3B1*(11): 5′-CCATGTGGTTTGCTGTTACCAA-3′ (forward) and 5′-TCAAAACGACCCTCAAGTTAAAAGA-3′ (reverse);

*GREB1:* 5′-GGGATCTTGTGAGTAGCACTGT-3′ (forward) and 5′-AATCGG TCCACCAATCCCAC-3′ (reverse);

*pS2:* 5′-GTCCCTCCAGAAGAGGAGTG-3′ (forward) and 5′-AGCCGA GCTCTGGGACTAAT-3′ (reverse);

*PGR*(39)*:* 5′- GTCGCCTTAGAAAGTGCTGTCAG-3′ (forward) and 5′-GCTTGGCTTTCATTTGGAACGCC-3′ (reverse);

*RPLP0* (large ribosomal protein P0, a housekeeping gene) (11): 5′-CGAGGGCACCTGGAAAAC-3′ (forward) and 5′-CACATTCCCCCG- GATATGA-3′ (reverse)

For steroid-treated cells, each mRNA transcript was quantified by normalizing the sample values to *RPLP0* and vehicle-treated cells. All gene expression studies were repeated in at least three independent experiments.

### ChIP analysis

ChIP analysis was performed using the SimpleChIP Plus Sonication Chromatin IP Kit (#56383, Cell Signaling Technology) according to the manufacturer's protocol. T47D cells were cross-linked with 1% formaldehyde for 10 min at room temperature, quenched with 0.125 M glycine, and lysed to isolate chromatin. Sonication was used to shear DNA to 200 to 500-bp fragments. Chromatin was immunoprecipitated overnight at 4 °C using an anti-LRH1 antibody (ABE2867, Millipore) or normal rabbit IgG (#2729, Cell Signaling Technology) as a negative control.

Immunoprecipitated DNA was analyzed by quantitative PCR using primers spanning the proximal HSD3B1 promoter region (−200–0 bp relative to the transcription start site). Enrichment was calculated using the 2ˆ−ΔCT method relative to vehicle-treated controls and expressed as percent input. Primer specificity was confirmed by evaluation of dissociation curves and by independently analyzing amplified product on an agarose gel.

The LRH1 binding motifs were predicted by JASPAR. Primers on the upstream of HSD3B1 translation start site were designed by NCBI primer design as below:

− *200∼0 Primer1*: 5′-GATGGGACTTCTCTTCCTGTTC-3′ (forward) and 5′-TCACTTTGATCTCTGGCCATGTA-3′ (reverse);
− *200∼0 Primer2:* 5′-CACTGCAGCATTAGGATGGGA-3′ (forward) and 5′-TGGCCCAACCCTTATCACTT-3′ (reverse);
− *200∼0 Primer3:* 5′-TTTGCCACACTGCAGCATTAG-3′ (forward) and 5′-CTTCTGGCCCAACCCTTATCA-3′ (reverse);
− *660∼-460:* 5′- GGCATACAACCACAAATCCCC-3′ (forward) and 5′- CCTCAATGGTCATTTGCCCC -3′ (reverse);

### Western Blot analysis

Protein lysates were prepared using RIPA buffer supplemented with protease and phosphatase inhibitors (Thermo Fisher Scientific). Protein concentration was determined using a BCA protein assay (Pierce Protein Research Products, Thermo Fisher Scientific). Protein, 30 μg to 50 μg, was separated by 10% SDS-PAGE gels and transferred to PVDF membranes (Millipore). After incubating overnight at 4 °C with the anti-3βHSD1 antibody, anti-NR5A2 antibody, anti-NR5A1 antibody, or anti-GAPDH antibody as appropriate, the appropriate secondary antibody was added and incubated for 1 h at room temperature, and signals were visualized using ECL detection reagent (Thermo Fisher Scientific). An anti-GAPDH antibody (1:5000; Sigma-Aldrich) was used as a control for sample loading.

### Steroid metabolism

Cells (600,000–800,000 cells per well) were seeded and maintained in 6-well plates that were coated with poly-L-ornithine (Sigma-Aldrich) for 12 h and then treated with [^3^H]-DHEA (100 nM, 300,000–600,000 cpm; PerkinElmer). Cells were incubated at 37 °C, and aliquots of medium (0.3 ml) were collected at the indicated time points. Collected media were incubated with 300 U β-glucuronidase (*Helix pomatia*; Novoprotein) at 37 °C for at least 2 h, extracted with 600 μl 1:1 ethyl acetate: isooctane, and concentrated under a nitrogen stream.

For high-performance liquid chromatography (HPLC) analysis, the concentrated samples were dissolved in 50% methanol and injected on a Breeze 1525 system equipped with a model 717 plus autoinjector (Waters Corp.). Steroid metabolites were separated on a Luna 150 × 4.6 mm, 3 μm C18 reverse-phase column (Phenomenex) using methanol/water gradients at 30 °C. The column effluent was analyzed using a β-RAM model 3 in-line radioactivity detector (IN/US Systems Inc.) with Liquiscint scintillation mixture (National Diagnostics). All metabolism studies were performed in triplicate and repeated in independent experiments.

### Cell viability assays

Cells (∼10^4^/well) were plated in triplicate in 96-well plates coated with poly-dl-ornithine, incubated overnight, then starved with phenol red–free medium containing 10% charcoal: dextran-stripped fetal bovine serum for 48 h and treated with 100 nM DHEA and combined with indicated drug treatments for indicated times, and assayed using the CellTiter-Glo Luminescent Cell Viability Assay (Promega). Absorbance was normalized to controls as indicated. For trypan blue staining, cells were incubated with 0.4% trypan blue (Sigma-Aldrich) and counted using a hemocytometer.

### Estrone (E1) and estradiol (E2) quantification (mass Spectrometry/ELISA)

#### Steroid extraction

Freshly collected media samples were frozen and kept at −80 ˚C until the LC/MS/MS analysis, as reported previously with slight modifications (40, 41). For the analysis, 250 μl media sample was spiked with 10 μl internal standards mix [5 ng/ml of E2-^13^C_3,_ 25 ng/ml, androstene-3, 17-dione-2,3,4-^13^C_3,_ and 5α-dihydrotestosterone-d3 (16,17,17-d3)] in a glass tube. The steroids were extracted using methyl-tert-butyl ether (MTBE, Across) using liquid-liquid extraction. The combined MTBE fractions were dried under a gentle nitrogen gas flow. Then the dried sample was reconstituted with 120 μl of 50% methanol [methanol/water (v/v)]. The reconstituted sample was divided into two fractions, one for estrogen and one for androgen analyses.

#### Estrogen analysis

An ultra-high-pressure liquid chromatography (NEXERA X2, Shimadzu Corporation) system with a C18 column (InfinityLab Poroshell 120 EC-C18 column, 4.6 × 75 mm, 2.7 μm, Agilent) and a gradient was used to separate estrogens in one of the prepared fractions. The separated estrogens were selected and quantified by mass spectrometry (Qtrap 6500+, AB Sciex) by using multiple reaction monitoring (MRM) mode in negative ion ESI. MultiQuant Software (version 3.0.3, AB Sciex) was used for data acquisition and quantification for estrogens and androgens.

For the LC-MS/MS method:

• **Estrone (E1):** LLOD = 1.25 pg/ml, LLOQ = 5 pg/ml. Intraday relative errors for standard spiked samples in CSS-FBS media were 12.99% (5 pg/ml), 1.14% (50 pg/ml), 6.57% (250 pg/ml), and 2.44% (500 pg/ml), with precision (CV) values ranging from 10.04% to 2.73%. Interday relative errors were 17.26%, 2.39%, 7.51%, and 1.25% for the same concentrations, with precision (CV) values between 13.45% and 5.53%.
• **Estradiol (E2):** LLOD = 2.5 pg/ml, LLOQ = 5 pg/ml. Intraday relative errors were 6.52% (5 pg/ml), 0.44% (50 pg/ml), 5.76% (500 pg/ml), and 2.78% (10,000 pg/ml), with corresponding precision (CV) values of 7.73%, 6.71%, 2.00%, and 0.84%. Interday relative errors were 15.92%, 1.35%, 5.07%, and 2.76%, with precision ranging from 16.24% to 0.75%.

Accuracy and precision were assessed using QC samples prepared by spiking CSS media with standards as detailed in Table S1.

### Clinical data analysis

To assess the clinical relevance of *HSD3B1* and *NR5A2*, we reanalyzed publicly available, processed RNA-seq data from hormone therapy–treated breast cancer patients enrolled in the POP trial (NCT02008734), as reported in the supplementary materials of Guerrero-Zotano et al. (32). The correlation between *HSD3B1* and *NR5A2* expression was determined using Spearman's rank correlation.

### Statistical analysis

All experiments were performed in triplicate, and data are presented as mean ± SEM. Statistical significance was determined using unpaired two-tailed Student's *t*-tests or one-way ANOVA in GraphPad Prism 9. A *p*-value < 0.05 was considered statistically significant.

## Data availability

All data needed to evaluate the conclusions in the paper are available in the main text or the supporting information. The processed RNA-seq data analyzed in this study were obtained from the supplementary materials of Guerrero-Zotano et al. (32).

## Supporting information

This article contains supporting information.

## Conflict of interest

The authors declare that they have no conflicts of interest with the contents of this article.
