# Supplementary material for: HSD3B1 upregulation via LRH1 sustains estrogen receptor signaling and promotes endocrine resistance in breast cancer[image]
Source: J Biol Chem. 2025 Jun 20;301(7):110405. doi: 10.1016/j.jbc.2025.110405 (PMC12281528; doi:10.1016/j.jbc.2025.110405)
Supplement: Supplementary table [file mmc1.docx]

**Supplementary Table 1.** Accuracy and precision for estrogens were assessed using QC samples prepared by spiking CSS media with standards. Each batch included two calibration curves, one at the beginning and one at the end, covering the following concentrations: 2.5, 5, 50, 250, and 500 pg/mL for E1, and 5, 50, 250, 500, and 10,000 pg/mL for E2. Ion transitions used were 269.2→144.9 and 269.2→143.1 for E1, and 271.1→183.0, 271.1→144.9, and 271.0→143.1 for E2. A ^13^C_3_-E2 was used for quantification. Retention times were 8.69 minutes for E1 and 8.47 minutes for E2 and ^13^C_3_-E2.

| Parameter | E1 | E2 |
| --- | --- | --- |
| LLOD (pg/mL) | 1.25 | 2.5 |
| LLOQ (pg/mL) | 2.5 | 5 |

| Analyte | Concentration (pg/ml) | Intraday (n=3) | | Interday (n=9) | |
| --- | --- | --- | --- | --- | --- |
|  |  | Relative Error (%) | Precision, CV (%) | Relative Error (%) | Precision, CV (%) |
| Estrone | 5 | 12.99 | 10.04 | 17.26 | 13.45 |
|  | 50 | 1.14 | 1.74 | 2.39 | 5.77 |
|  | 250 | 6.57 | 4.15 | 7.51 | 6.55 |
|  | 500 | 2.44 | 2.73 | 1.25 | 5.53 |
| Estradiol | 5 | 6.52 | 7.73 | 15.92 | 16.24 |
|  | 50 | 0.44 | 6.71 | 1.35 | 8.82 |
|  | 500 | 5.76 | 2.00 | 5.07 | 2.05 |
|  | 10000 | 2.78 | 0.84 | 2.76 | 0.75 |
