# Supplementary material for: HSD3B1 upregulation via LRH1 sustains estrogen receptor signaling and promotes endocrine resistance in breast cancer[image]
Source: J Biol Chem. 2025 Jun 20;301(7):110405. doi: 10.1016/j.jbc.2025.110405 (PMC12281528; doi:10.1016/j.jbc.2025.110405)

***HSD3B1* Upregulation via LRH1 Sustains Estrogen Receptor Signaling and Promotes Endocrine Resistance in Breast Cancer**

**Xiuxiu Li1, Yoon-Mi Chung1, Monaben Patel2, Nima Sharifi1,3*.**

^1^ Desai Sethi Urology Institute, Sylvester Comprehensive Cancer Center, University of Miami Miller School of Medicine, Miami, FL 33136, USA. ^2^Lerner Research Institute, Cleveland Clinic, Cleveland, OH 44195, USA. ^3^Lead contact

* Corresponding author

Nima Sharifi

Desai Sethi Urology Institute, Sylvester Comprehensive Cancer Center, University of Miami Miller School of Medicine, Miami, FL 33136, USA

Email: [nimasharifi@miami.edu](mailto:nimasharifi@miami.edu)

Running title: *HSD3B1* Promotes Endocrine Resistance in Breast Cancer

Keywords: *HSD3B1,* estrogen synthesis, Endocrine Resistance, Breast Cancer

**Supplemental Figure Legends**

**Figure S1**. (A) MCF7 cells were cultured in 10% charcoal-stripped serum (LTED) DMEM medium or treated with 2 μM 4-hydroxy tamoxifen (4OHT) for 21 days, *HSD3B1* mRNA levels were assessed by qPCR and protein levels were assessed by Western blot. Data represent the mean ± SEM of three independent experiments.(B) Long-term aromatase inhibitor treatment increases *HSD3B1* expression in T47D cells. T47D cells were treated with 10 μM letrozole for 35 days, followed by qPCR analysis of *HSD3B1* expression. Data represent the mean ± SEM of three biological triplicates. (C)(D) T47D cells were cultured in 10% charcoal-stripped serum (LTED) RPMI-1640 medium or treated with 2 μM 4-hydroxy tamoxifen (4OHT) for 21 days, *ESR1, ESR2* and *CYP19A1* mRNA levels were assessed by qPCR and protein levels were assessed by Western blot. Data represent the mean ± SEM of three independent experiments. * *P* < 0.05. ** *P* < 0.01.*** *P* < 0.001.


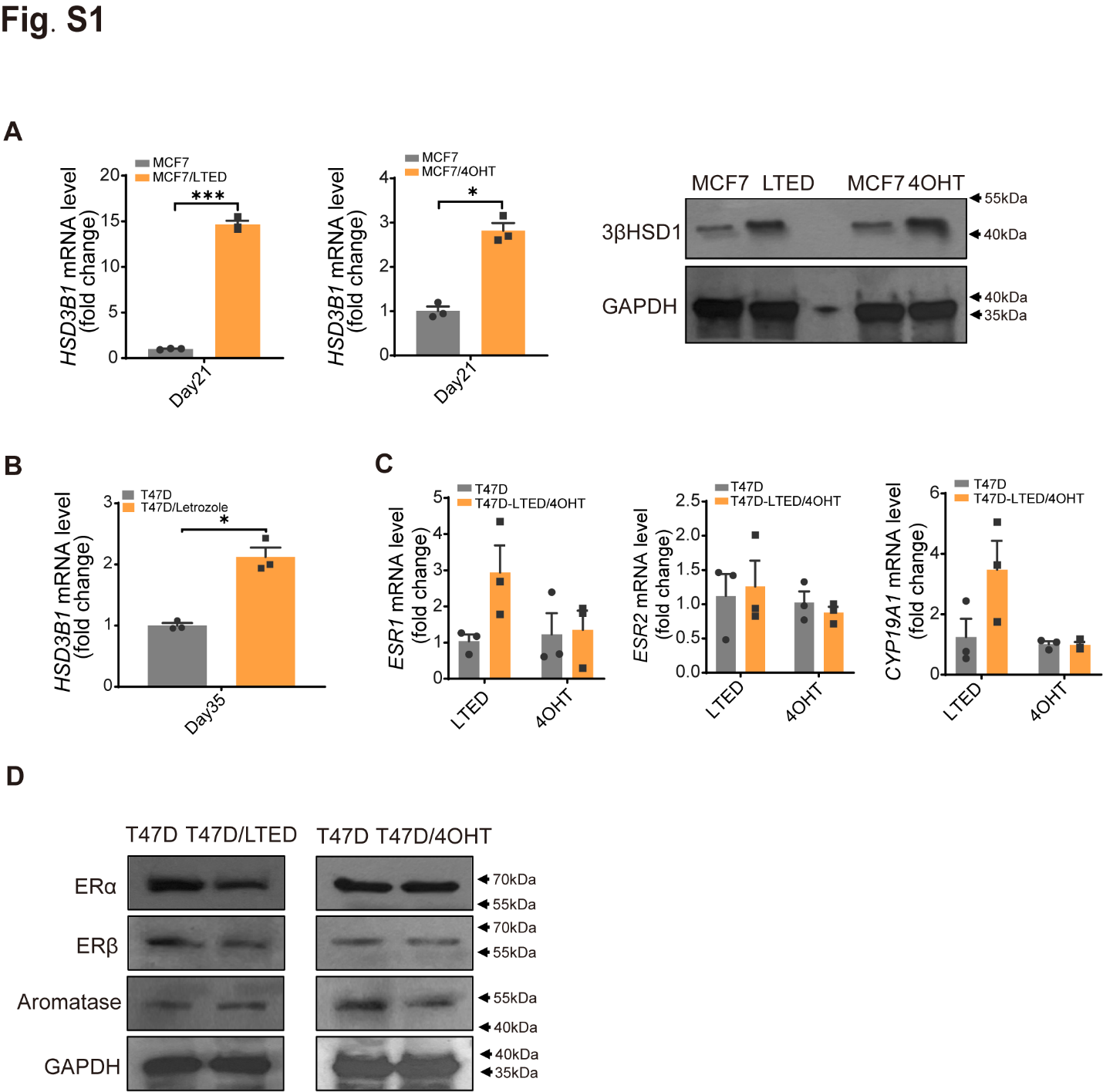


**Figure S2**. (A) T47D cells were cultured in 10% charcoal-stripped serum (LTED) RPMI-1640 medium or treated with 2 μM 4-hydroxy tamoxifen (4OHT) for 21 days, LRH1 protein level was assessed by Western blot. (B) MCF7 cells were treated with LRH1 inhibitor for 48 hours, followed by qPCR and Western blot analysis of *HSD3B1* expression. Data represent the mean ± SEM of three biological triplicates. (C) MCF7 cells were transfected with an *NR5A2* expression construct or empty vector control for 48 hours, followed by qPCR and Western blot analysis of *HSD3B1* expression. Data represent the mean ± SEM of three biological triplicates. (D) T47D cells were transfected with an *NR5A2* expression construct or empty vector control for 48 hours, followed by qPCR and Western blot analysis of *HSD3B1* expression. Data represent the mean ± SEM of three biological triplicates. (E) Chromatin immunoprecipitation (ChIP) followed by qPCR was performed using an LRH1-specific antibody in T47D cells. Enrichment of LRH1 at the HSD3B1 promoter (−200 to 0 bp) was quantified using primers spanning the proximal promoter region. Two high-scoring LRH1 motifs (AACCCAAAGGTCACT and GAGTACATGGCCAGA) were predicted in the −130 to −60 bp region using the JASPAR database. Data represent the mean ± SEM of three independent experiments. * *P* < 0.05. ** *P* < 0.01.*** *P* < 0.001.


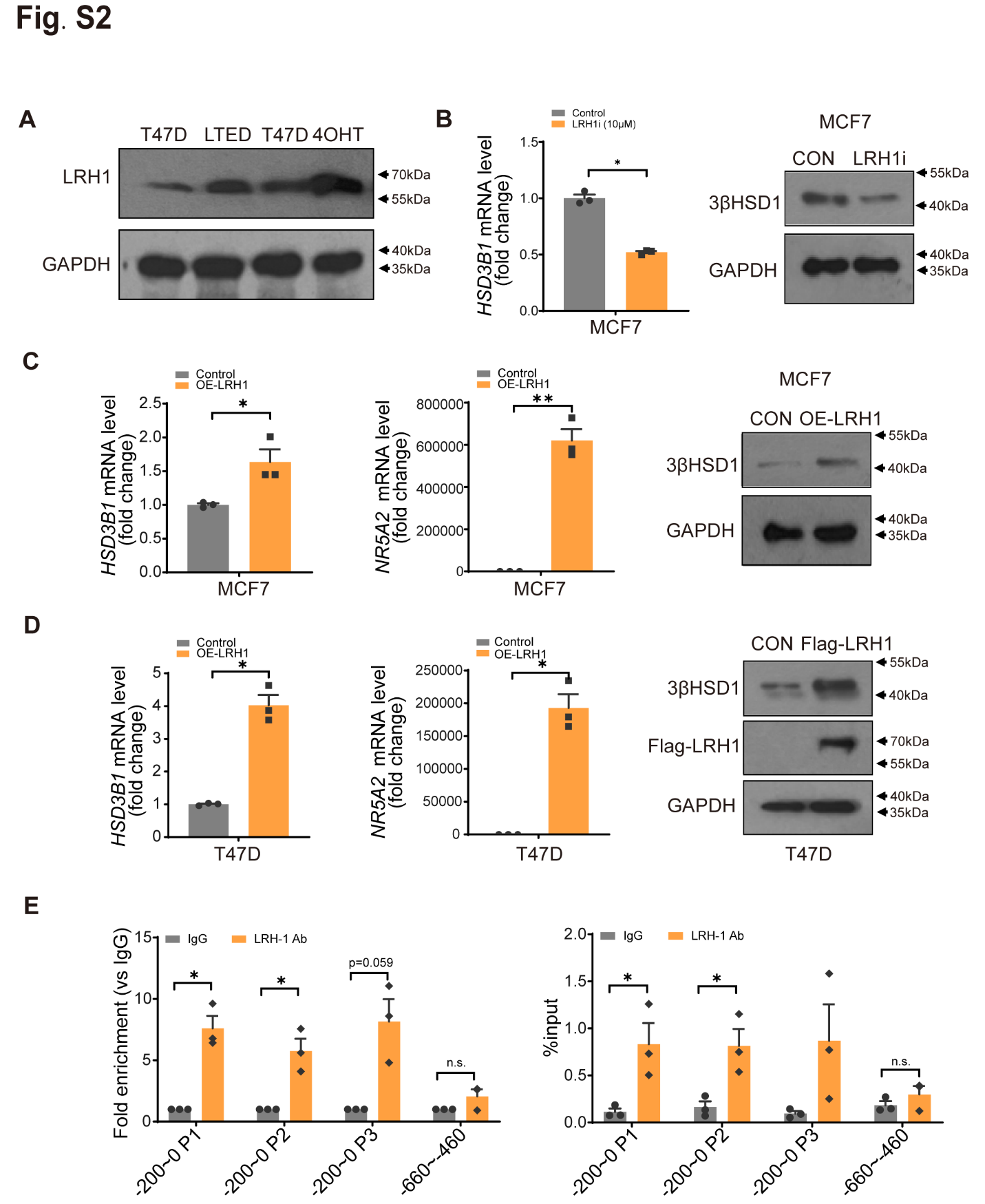


**Figure S3**. (A) T47D-4OHT cells were treated with SF1 or LRH1 inhibitors for 12 hours, followed by [³H]-DHEA metabolism analysis by HPLC. (B) Stable T47D cell lines with *NR5A2* gRNA or control gRNA were generated, and LRH1 protein expression was analyzed by Western blot. (C) NR5A2 gRNA or control T47D cell lines were cultured with 2 μM 4OH-tamoxifen for 35 days, then treated with [³H]-DHEA, and steroid metabolism was assessed by HPLC. Data represent the mean ± SEM of three independent experiments. * *P* < 0.05. ** *P* < 0.01.*** *P* < 0.001.


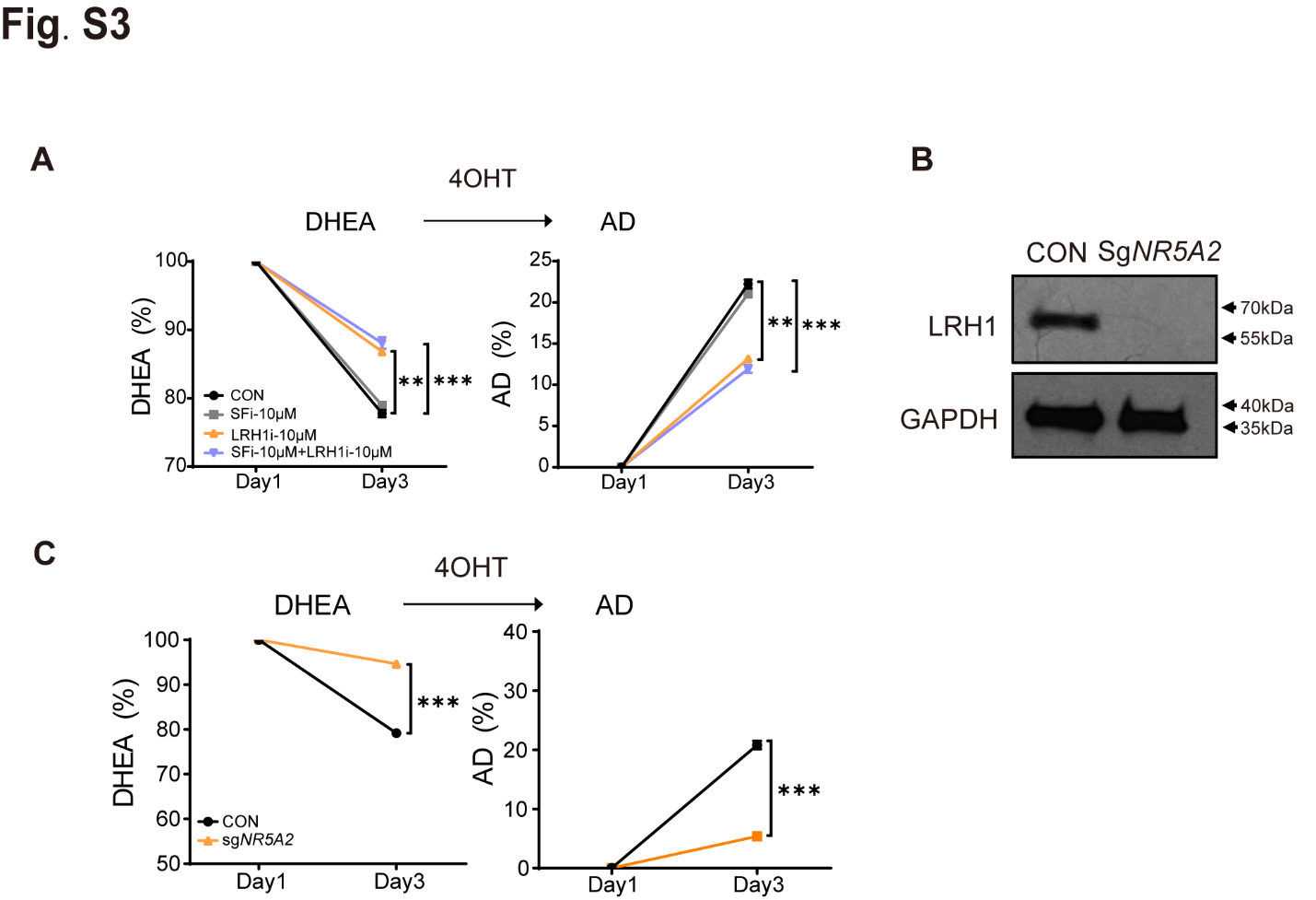


**Figure S4**. (A) T47D-LTED and T47D-4OHT cells were treated with SF1 or LRH1 inhibitors for 12 hours, followed by 40nM Estradiol (E2) treatment for 7 days, and qPCR analysis of ER target gene expression. (B) NR5A2 gRNA or control T47D cell lines were cultured in RPMI-1640 medium containing 10% charcoal-stripped fetal bovine serum for 21 days, followed by 40nM Estradiol (E2) treatment for 7 days, and qPCR analysis of ER target gene expression. (C) (D) T47D-LTED and T47D-4OHT cells were pre-treated with SF1 or LRH1 inhibitors for 12 hours, followed by with or without 40nM Estradiol (E2) treatment for indicated days, and cell viability was assessed. (E) NR5A2 gRNA or control T47D cell lines were cultured in RPMI-1640 medium containing 10% charcoal-stripped fetal bovine serum for 21 days, followed by 40nM Estradiol (E2) treatment for indicated days, and cell viability was assessed. (F) T47D cells stably expressing lenti-NR5A2-FLAG were cultured in RPMI-1640 medium supplemented with 10% charcoal-stripped fetal bovine serum for 2 days to deplete endogenous hormones. Cells were then treated with DHEA for the indicated days. Cell viability was assessed, and expression of estrogen receptor (ER) target gene was analyzed by qPCR. Data represent mean ± SEM from three independent biological replicates.Data represent mean ± SEM from three independent biological replicates (unpaired two-tailed t-test). * *P* < 0.05. ** *P* < 0.01.*** *P* < 0.001.


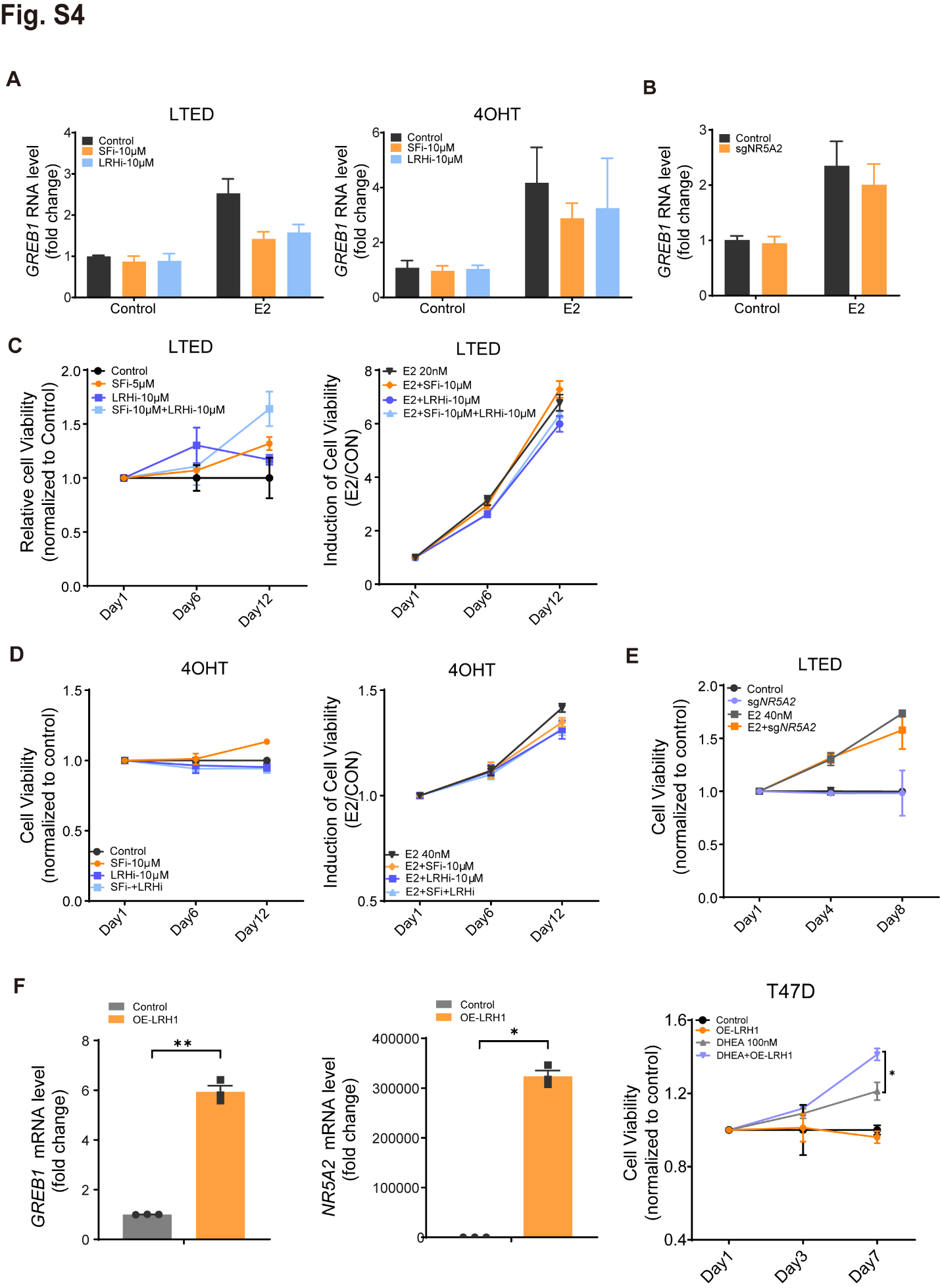

Supplement: Supplementary figures [file mmc2.docx]
